# Music Therapy for People With Autism Spectrum Disorder: A Systematic Review of Randomized Clinical Trials

**DOI:** 10.7759/cureus.81361

**Published:** 2025-03-28

**Authors:** Mazen Alayidh, Feras Alawad, Wateen B Alanazy, Fatimah O Al-Harbi, Abdulelah M Alotaibi, Qonoot A Al Mohammed, Abdullah S Aljubran, Reem T Al-Otaibi, Raghad F Al-Otaibi, Tariq R Al Rubaie

**Affiliations:** 1 College of Medicine, King Khalid University, Abha, SAU; 2 Department of Psychiatry, Imam Abdulrahman Bin Faisal University, Dammam, SAU; 3 College of Medicine, Al Jouf University, Sakaka, SAU; 4 College of Pharmacy, Taibah University, Madinah, SAU; 5 College of Medicine, Taif University, Taif, SAU; 6 College of Nursing, King Saud bin Abdulaziz University for Health Sciences, Al-Ahsa, SAU; 7 College of Medicine, Imam Abdulrahman Bin Faisal University, Dammam, SAU; 8 College of Applied Medical Sciences, Clinical Laboratory Sciences, Shaqra University, Shaqra, SAU; 9 College of Applied Medical Sciences, Taif University, Taif, SAU

**Keywords:** asd, autism spectrum disorder, music therapy, rct, systematic review

## Abstract

Autism spectrum disorder (ASD) affects social interaction, communication, and learning, with its prevalence continuing to rise, and music therapy (MT) has shown promise in improving social interactions and communication skills in individuals with ASD. This systematic review explores the relationship between ASD and music therapy, examining factors that influence its effectiveness in children. A systematic review of randomized controlled trials (RCTs) published between 2009 and 2024 was conducted to assess the effects of music therapy on children with autism. Studies were retrieved from databases such as PubMed and Google Scholar, with the final search completed by October 1, 2024, and only RCTs that evaluated music's impact on ASD and reported relevant outcomes were included, while non-RCTs, studies with a high risk of bias, and duplicates were excluded. A total of nine RCTs involving 1,327 children with ASD, aged 2-12, were analyzed; these studies assessed various music therapy interventions lasting from two weeks to eight months, with sessions occurring one to three times per week. Findings were mixed, as four studies with 449 participants reported significant improvements in social communication skills, while three larger studies with 715 participants found no significant changes in primary social outcomes but noted improvements in specific aspects of social responsiveness, and two smaller studies with 59 participants reported notable enhancements in verbal production and emotional responsiveness. Music therapy has been recognized as a beneficial intervention for improving health outcomes across various conditions, including mental health disorders, and this review highlights its potential in autism, particularly in enhancing cognitive processing, emotional responses, and social communication; however, while the findings are promising, further research with larger sample sizes and extended study durations is necessary to validate these effects.

## Introduction and background

Autism spectrum disorder (ASD) is a neurological and developmental condition affecting social interaction, communication, and learning. It is categorized as a "developmental disorder," diagnosable at any life stage, with most symptoms appearing within the first two years. According to the Diagnostic and Statistical Manual of Mental Disorders, Fifth Edition (DSM-5), people with ASD often struggle with communication and social interaction, have restricted interests, and exhibit repetitive behaviors, impacting their functioning in various life areas [1]. Recent studies indicate a significant rise in ASD prevalence, estimated at one in 1,000 individuals [2], with males being affected more frequently than females (ratio: 4.3:1, varying with mental retardation status) [3]. Music therapy (MT) is an established healthcare practice that harnesses the power of music in a clinical setting to address the physical, emotional, cognitive, and social needs of individuals. Through music, therapists can target issues related to mobility, perception, and emotions, effectively addressing the challenges faced by individuals, including children with significant physical, intellectual, or emotional difficulties [4]. In 2007, global research showed some findings indicating that long-term active music therapy (AMT) may be beneficial in treating clinical symptoms and musical abilities among young adults with severe autism. AMT sessions may enhance autistic symptoms and personal musical skills, potentially due to the high level of absorption and personal interaction involved in musical engagement [5]. Studies have shown MT's effectiveness in improving social interaction and communication skills in individuals with ASD. One study found associations between age, receptive vocabulary, and emotion recognition abilities [6]. Another indicated that the therapeutic relationship in MT predicts clinical changes in ASD symptom severity, emphasizing the importance of therapist attunement to the child's communication style [7]. Some research provides preliminary evidence for MT's potential in improving emotion recognition, reducing alexithymia symptoms, and enhancing clinical symptoms and musical skills in individuals with ASD across different age groups. Improvisational MT, particularly child-centered methods, appear to have potential benefits for social-emotional and motivational growth [8].

Compared to toy play, MT produced more events of joy, emotional synchronicity, and engagement initiation, with stronger effects in child-led sessions. The therapeutic relationship in MT is suggested as an important predictor of outcomes, particularly for communication and language skills [9].

However, many studies have had small sample sizes and varied in outcome measures and intervention protocols. Future research should employ a more standardized and controlled approach to overcome the limitations of prior research. The main objective of this study is to assess the relationship between autism spectrum disorder (ASD) and music therapy and to address the factors influencing this relationship among children with ASD.

## Review

Methods

Search Strategy

The researchers conducted a literature review following the Preferred Reporting Items for Systematic Reviews and Meta-Analyses (PRISMA) statement guidelines. This systematic review, registered at PROSPERO under number CRD42024571336, demonstrates our commitment to transparency and best practices.

The article was done without conducting a meta-analysis due to significant clinical heterogeneity among the included studies. Variability in patient populations, interventions, and outcomes made it inappropriate to statistically pool the results. Additionally, inconsistencies in data reporting and the small number of studies addressing certain outcomes also contributed to this decision. Instead, a narrative synthesis was conducted to summarize the evidence while maintaining transparency and accuracy in interpreting the findings.

This approach, which is known for its rigor and focus, was used to identify, screen, and analyze relevant studies. The search was further refined using specific search algorithms, ensuring that only the most relevant and up-to-date literature was included. Two electronic databases, PubMed and Google Scholar, were searched, with the search process concluding on October 1, 2024.

The PubMed search was carefully constructed to ensure the significance of the results. Key search terms such as "Music Therapy"[MeSH], "music therapy," "music intervention," "Autism" [MeSH], "autism spectrum disorder," "ASD," and "Randomized Controlled Trial" [Publication Type], "randomized controlled trial," "randomized clinical trial," "RCT," "Systematic Review" [Publication Type], "systematic review," and "English" [Language] were carefully selected for their relevance and accuracy.

The search phrases for Google Scholar included "music or music therapy" and "autism spectrum disease, autism, or ASD."

Inclusion and Exclusion Criteria

The study included any research that met the following criteria: studies published between 2009 and 2024, studies investigating the effects of music on patients with autism, studies published in the English language, inclusion of randomized controlled trials, and studies reporting outcomes relevant to music therapy in autism spectrum disorder. These criteria are set to ensure a comprehensive and focused review.

The study excluded research that does not report relevant outcomes, studies published in languages other than English, studies that are not randomized controlled trials, studies with a high risk of bias or low quality, and duplicates.

Study Screening and Data Extraction

The screening process involved a thorough and comprehensive evaluation of titles and abstracts and a detailed review of the full text from each database to determine eligibility. The assessment included a screening of titles and abstracts, with all articles being carefully screened and subsequently categorized as either "included," "excluded," or "undecided." The titles and abstracts of all identified articles underwent independent screening by four individuals, with conflicts resolved by two authors. Six authors participated in the screening process based on title and abstract. Subsequently, another screening phase was conducted involving the full text to ensure the inclusion of all available articles. Articles were included if they described the methodology and measurable results or outcomes and if they were compared against specified inclusion and exclusion criteria, which were thoughtfully designed to ensure the methodological precision of the study.

An electronic summary was generated using an Excel program to create a table that extracted data from all included studies. This extraction was conducted based on the research findings, ensuring that the data was not only comprehensive but also relevant and applicable to the field of music therapy. The criteria for extraction included DOI, title, author, year, journal, region, study design, duration, sample size, age range, distribution, diagnosis details, type of music therapy, duration and frequency of therapy, control, materials, measurement tools, outcomes, risk of bias, blinding, randomization, engagement level, and limitation.

Risk of Bias Assessment

The risk of bias in the included studies was assessed using the Cochrane Risk of Bias tool 2 (RoB 2). The authors independently evaluated the included studies' methodological quality. Disagreements were resolved by consulting with additional authors.

We evaluated the following items: random sequence generation, allocation concealment, blinding of participants and personnel, blinding of outcome assessment, incomplete outcome data, selective reporting, and other biases for each trial. Based on the data presented in the review, each item's risk of bias was assessed as low, high, or unclear.

Results

**Figure 1:**
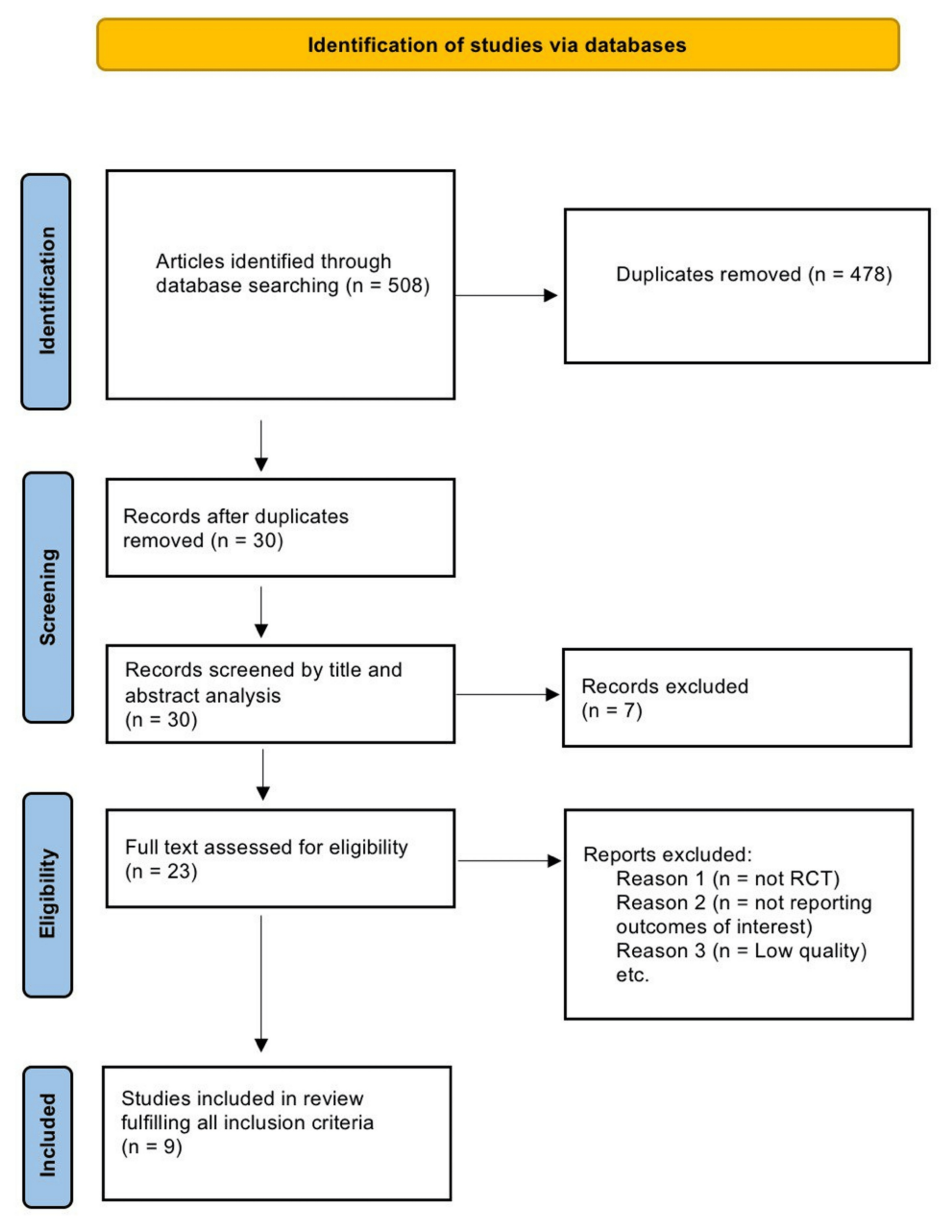
PRISMA flowchart showing the article selection process for nine chosen articles PRISMA: Preferred Reporting Items for Systematic Reviews and Meta-Analyses, RCT: randomized controlled trial

The review provides an overview of nine RCTs into the differential application effects music therapy has on patients with autism. A comprehensive search for articles was conducted covering the period from 2009 to 2024. Figure 1 presents a flow diagram showing the steps followed in the process of selecting research to identify eligible papers. The targeted outcomes were to be based on social communication, emotional responsiveness, and behavioral improvements. The main findings from such studies are summarized below, listed according to the primary outcomes measured.

Social Communication and Responsiveness

Several studies have focused on the impacts of music therapy in developing a child who has ASD in terms of social communication and responsiveness.

Williams et al. (2024) investigated the efficacy of a Music-Assisted Program (MAP) in enhancing social responsiveness and language development in autistic children aged 2-5 years. In this study, there was an 18-week intervention with two sessions per week. The MAP was compared with the Social Communication Intervention for Preschoolers-Intensive (SCIP-I). Although the estimation of effect sizes for future research was the primary aim of the study, the preliminary findings indicated that the MAP participants had greater improvements in social responsiveness and vocabulary than the SCIP-I group; however, specific effect sizes were not calculated [10].

Sharda et al. (2018), for instance, carried out an experiment involving 51 kids between the ages of six and 12 years. The children were involved in a music-based intervention once a week for 8-12 weeks for comparison with those undergoing non-music interventions. This was measured using the Children's Communication Checklist - Second Edition (CCC-2) and the Social Responsiveness Scale, Second Edition (SRS-II); significant results were observed in this aspect. The mean difference in music treatment in social communication scores between groups was 4.84, hence being more advantageous in this approach. Further, brain connectivity analyses demonstrated that the resting-state functional connectivity (RSFC) of frontotemporal networks featured significant increases, with z-scores as high as 3.94 and 3.16, indicating that, in fact, the intervention may have beneficial influences on both behavior and the brain [11].

Geretsegger et al. (2011) investigated the social communication skills of three hundred children aged between four and seven years of age as a function of the effects of improvisational music therapy (IMT). Participants were randomly assigned to either the IMT group or to the control group, which received standard care. Sessions were held once or three times per week over the five months that the intervention was in place. Although there were no significant differences between groups in primary outcome measures using the Autism Diagnostic Observation Schedule social affect scores, there were significant improvements in social engagement and responsiveness in the IMT group; the high-frequency sessions yielded the greatest gains, especially twice a week [12].

Bieleninik et al. (2017), in their international multicenter RCT, compared Improvisational Music Therapy (IMT) plus enhanced standard care (ESC) against ESC alone. A total of 364 children between the ages of four and seven were engaged in the trial. The primary outcome measured by the Autism Diagnostic Observation Schedule (ADOS) presented no detectable differences between groups in social affect scores. In contrast, several Social Responsiveness Scale (SRS) subscales, especially motivation and social communication, revealed small but statistically significant gains [13].

The multicenter trial of Crawford et al. (2017) tested 364 children who were 4-7 years of age and showed limited overall improvement in social affect scores. However, slight gains in social responsiveness were observed in secondary measures. Overall, the benefits of IMT, compared to enhanced standard care alone, may emerge only under specific circumstances or for specific subgroups of children [14].

Green et al. (2010) tested whether or not the Preschool Autism Communication Trial (PACT) affected the social interactions and behaviors of 152 children aged 2-4 years and 11 months with ASD diagnosis. The intervention for this study consisted of a 12-month-long study: a regular six-month session phase at the very beginning was followed by a six-month follow-up. The intervention group, therefore, participated in the parent-mediated communication-focused therapy program, PACT, while the control group received treatment as usual (TAU). In the PACT group, a moderate effect size (Cohen's d = 0.64) was realized in the significant improvements in ADOS scores compared to those in the control group. These findings provide evidence that the PACT intervention supports young children with ASD in developing their social communication skills [15].

Emotional and Motivational Responsiveness

Kim et al. (2009) investigated the effects of IMT on emotional and motivational behaviors and hypothesized that IMT would enhance factors such as emotional involvement and peer motivation in 10 children with autism between the ages of 39 and 71 months. This randomized controlled trial conducted weekly 30-minute sessions for a total duration of 12 weeks. Sessions were completed in which child-led and therapist-led IMT was compared to IMT presented during toy play [9]. Results showed that music therapy conditions compared to toy play significantly increased emotional synchrony, joy, and initiation of engagement, which would indicate that IMT could be effective in enhancing emotional responsiveness and social motivation among young children with ASD [16].

Behavioral Improvements and Cognitive Functioning

Music therapy's impact on behavioral improvements and cognitive functioning was another significant focus across several studies.

Rabeyron et al. (2020) conducted an RCT with 37 children aged 4-7 years to compare music therapy (MT) to music listening (ML) over an eight-month period of 25 sessions. The study reported that, in comparison with the baseline assessment, the Clinical Global Impression (CGI) score increased significantly for the MT group, which reflects a large effect size (Cohen's d = 0.80). Besides, the lethargy and stereotypy subscales in the Aberrant Behavior Checklist (ABC) yielded significant improvements for the MT group, thus indicating that MT contains specific behaviorally active benefits [17].

Lim and Draper (2011) investigated the efficiency of the addition of a music therapy intervention to an Applied Behavior Analysis Verbal Behavior (ABA VB) approach on 22 three- to five-year-old children. The treatment was specifically focused on items targeting verbal operants: mand, tact, echoic, and intraverbal skills. These gains in the music therapy verbal operant productions proved to be significant when compared to both the standard ABA VB approach and no training. Music therapy was especially effective at enhancing echoic production, thus showing its potential for augmenting traditional behavioral therapy [18].

Discussion

Unlike previous reviews, this review systematically summarizes the findings of nine different music therapy interventions and their impacts on patients with autism. The various studies studied a number of outcomes, including social communication, emotional responsiveness, and improvement in behavior. The findings provide us with a more refined understanding of the various domains in which music therapy may be beneficial for individuals with autism spectrum disorder. A summary of the nine RCTs is shown in Table 1.

**Table 1:** Summary of the nine randomized controlled trials assessing the effects of various music therapy interventions on autistic patients, detailing participant age ranges, sample sizes, session duration and frequency, primary outcome measures, and reported significant findings for each intervention compared to controls MT: music therapy, ML: music listening, CGI: Clinical Global Impression, CARS: Childhood Autism Rating Scale, ABC: Aberrant Behavior Checklist, ABA VB: Applied Behavior Analysis Verbal Behavior, ASD: autism spectrum disorder, MAP: Music-Assisted Program, SCIP-I: Social Communication Intervention for Preschoolers-Intensive, IMT: Improvisational Music Therapy, ESC: Enhanced Standard Care, ADOS: Autism Diagnostic Observation Schedule, SRS: Social Responsiveness Scale, CCC-2: Children's Communication Checklist - Second Edition, SRS-II: Social Responsiveness Scale, Second Edition, RSFC: resting-state functional connectivity, PACT: Preschool Autism Communication Trial, SCQ: Social Communication Questionnaire

Study number	First author (year)	Intervention	Comparator	Age range	Sample size	Session duration and frequency	Primary outcome measures	Significant findings
1	Rabeyron et al. (2020) [17]	MT	ML	4-7 years	37 (19 in MT, 17 in ML)	30 minutes, 25 sessions over 8 months	CGI, CARS, ABC	Significant improvement in CGI and ABC scores in MT group; large effect size (Cohen's d = 0.80) for CGI improvement.
2	Lim and Draper (2011) [18]	MT with ABA VB approach	Standard ABA VB approach and no training	3-5 years	22 children with ASD	Varied, minimum of 3 days a week for 2 weeks	Verbal operant production (mand, tact, echoic, and intraverbal)	Significant improvements in verbal operant production for music versus no training conditions; music is the most effective for echoic production.
3	Williams et al. (2024) [10]	MAP	SCIP-I	2-5 years	27 (13 in MAP, 14 in SCIP-I)	45 minutes, 2 sessions per week for 18 weeks	Social responsiveness, expressive/receptive vocabulary, parent-child interactions	The MAP group showed greater improvement in social responsiveness and vocabulary than the SCIP-I group; effect sizes not calculated.
4	Kim et al. (2009) [9]	IMT	Toy play sessions	3-5 years	10 children with ASD	30 minutes, weekly for 12 weeks	Emotional and motivational responsiveness, interpersonal responsiveness	Significant improvements in emotional responsiveness, initiation of engagement, and motivational behaviors in the IMT group.
5	Crawford et al. (2017) [14]	IMT	ESC	4-7 years	364 participants (182 in each group)	30-45 minutes, once or thrice weekly for 5 months	Social affect (ADOS), social responsiveness (SRS)	No significant improvement in primary outcomes; small effects found in some secondary SRS subscales.
6	Sharda et al. (2018) [11]	Music-based intervention	Non-music intervention	6-12 years	51 children (26 in music group, 25 in non-music group)	Weekly sessions for 8-12 weeks	Social communication (CCC-2, SRS-II), brain connectivity (RSFC)	Significant improvements in social communication and brain connectivity; mean difference in social communication scores of 4.84.
7	Bieleninik et al. (2017) [13]	IMT with ESC	ESC	4-7 years	364 participants (182 in each group)	30-45 minutes, once or thrice weekly for 5 months	ADOS social affect, SRS	No significant differences in primary outcome (ADOS social affect); nominally significant effects in some SRS subscales.
8	Green et al. (2010) [15]	Structured music therapy program	Standard care	2-4 years and 11 months	152 children, 77 assigned to PACT; 75 to treatment as usual	Biweekly 2-hour clinic sessions for 6 months, followed by monthly booster sessions for 6 months (total 18 sessions)	SCQ	Significant improvement in social communication skills in the music therapy group compared to control; moderate effect size.
9	Geretsegger et al. (2011) [12]	IMT	Standard care	4-7 years	300 participants (150 in standard care and 75 in each type of music therapy)	Once or thrice weekly for 5 months	Social communication skills (ADOS), social engagement	Improvement in social communication skills in high-frequency IMT sessions compared to standard care; effect size not provided.

Social Communication and Responsiveness

Music therapy has immense potential to promote levels of social communication and responsiveness among ASD children. The studies of Sharda et al. (2018) [11] and Geretsegger et al. (2011) [12] assured strong evidence favoring music-based interventions in improving the skills of social communication.

In the RCT conducted by Sharda et al. (2018), 51 children aged 6-12 years were given an 8-12 week music-based intervention against a non-music intervention. Indeed, music therapy does seem to facilitate both social skills and neuroplastic changes in the sense that there was a great improvement in the mean difference in social communication scores of 4.84, with a greatly increased RSFC in frontotemporal brain networks. Since music therapy necessitates multiple parts of the brain responsible for social cognition and interaction, it may facilitate broader development in social communication. These dual benefits, behavioral and neural, suggest that music therapy may have this effect [11].

Similarly, a large sample of 300 children between the ages of four and seven was studied by Geretsegger et al. to determine how IMT affected their social communication skills. Although the primary social affect scores, as measured by ADOS, did not reach significance, the IMT group showed significant gains in social engagement and responsiveness. This was especially true for high-frequency sessions (twice a week). Music therapy could significantly enhance interactive behaviors and social motivation, essential elements in successful communication, but it cannot change the core symptoms as measured by standardized diagnostic instruments [12]. A study by Green et al. (2010) using the parent-mediated communication-focused PACT, with a moderate effect size (Cohen's d = 0.64), significantly enhanced social interactions and behaviors. The findings of this study, therefore, strengthen the message of the importance of including the caregivers in the therapeutic process and point to the possibility that parent education or mediation in music therapy treatment may strengthen the benefits on social communication skills [15].

In this regard, Bieleninik et al. (2017) [13] and Crawford et al. (2017) [14] have explored the outcomes of social communication. However, no clinically significant benefits were established in terms of primary outcome measures administered via ADOS social affect scores. The silver lining is that both studies have reported small but statistically significant benefits in a number of subscales related to SRS that, once again, might suggest an edge over particular domains such as motivation and social communication.

These findings highlight the need to investigate a broad range of outcomes beyond primary measures when trying to detect clinically relevant subtle changes. Evidence of the effectiveness of music therapy in enhancing the social communication and responsiveness of children with ASD is thus cumulative; this is especially so when the intervention is sufficiently intensive and involves major social agents, such as the parents. Such variability in findings may relate to differences in types, modes of delivery, and participant characteristics; thus, approaches need to be individually adapted to maximize benefits.

Emotional and Motivational Responsiveness

Studies have also shown that music therapy enhances emotional and motivational responsiveness among individuals with autism. In instances where Improvisational Music Therapy sessions replace toy play, as indicated by Kim et al. (2009), there are significant enhancements in emotional synchronicity, joy, and the initiation of engagement. While both therapist-led and child-led sessions were effective in promoting unstructured emotional expression, the latter was slightly more effective. These findings indicate that young children with ASD can significantly benefit from music therapy in terms of increased emotional responsiveness and social motivation, especially if the treatment encourages child autonomy and active participation. The results of Kim et al. (2009) are corroborated by the extant literature with respect to the efficiency of music therapy in improving emotional involvement [9].

Music therapy may directly access emotional and motivational systems and transcend language difficulties, providing a non-verbal channel for communication and expression, with parallel enhancements in the potential for more natural and spontaneous social contact. As such, this approach can be so much more effective for children with ASD, who may hardly benefit from conventionally verbally mediated therapies [16].

Behavioral Improvements and Cognitive Functioning

Other studies also primarily focused on the impacts of music therapy on behavioral improvements and cognitive functioning. In these respects, children who received music therapy showed significant behavioral improvements as compared to those who participated in music listening alone [17]. The large effect size revealed for CGI scores and gains on the lethargy and stereotypy subscales of the ABC, extending over an eight-month period with 25 sessions in the present study, was Cohen's d = 0.80. It suggests that active music therapeutic participation is associated with significant decreases in maladaptive behaviors and increases in general functioning. Likewise, Lim and Draper (2011) investigated the usage of music therapy and the ABA VB method in augmenting verbal operant production in young children [18].

Music might also be an effective augmentation to the more typical behavioral therapies in providing a variety of added stimuli that help in learning and acquiring skills, as evidenced by the significant increase of mand, tact, echoic, and intraverbal skills of participants under the music therapy condition in this study. Notably, the increase in echoic production for the music therapy condition would suggest that musical elements, such as rhythm and melody, may facilitate verbal imitation and repetition, an important component in early language development [17,18]. The study by Crawford et al. (2017) into Improvisational Music Therapy combined with ESC showed no significant differences in the primary outcomes of social affect scores, although there were nominal improvements in secondary measures of social responsiveness. These results would support that, although possibly not greatly enhancing the primary symptoms of autism, music therapy may be effective for certain behavioral and cognitive domains, particularly when it is combined with comprehensive care approaches [14].

Limitations

Although this systematic review has highlighted the efficacy of music therapy for individuals with ASD, there are a number of limitations that have to be considered. First, the studies included were very heterogeneous regarding intervention types, frequencies of sessions, and measures of outcomes, which prevented the identification of a standard approach. The range of methodologies, from improvisational to structured interventions, complicates direct comparisons and limits the ability to generalize results.

Furthermore, several studies had small sample sizes, reducing statistical power, and many lacked long-term follow-up to determine the durability of observed effects. In some studies, the use of subjective or parent-reported measures may introduce bias, whereas objective measures such as neuroimaging were less frequently used. Finally, cultural and contextual factors varied across studies, which influenced the generalizability of results.

General benefits and effectiveness

In general, evidence supports music therapy as a promising intervention for individuals with ASD, given that the treatment is of sufficient intensity and tailored according to their needs. From social communication and emotional involvement to behavioral control and cognitive processing, this is a multilevel area of potential benefits that are holistic in the support of development in autistic individuals. Future studies should continue to refine these interventions, exploring optimal modes of delivery, frequencies, and intensities, besides determining which subgroups respond best, to maximize the therapeutic outcome.

## Conclusions

Music therapy (MT) is an intervention that has been widely used to enhance health outcomes for individuals with many different conditions, including mental health disorders. This study aimed to assess the impact of music therapy on autism patients by reviewing recent research. Some of the studies included in this review evaluated social communication skills among autism patients. The results consistently indicated that music therapy affected not only social communication and responsiveness but also emotional and motivational behaviors. Although the different types of interventions yielded varying outcomes, the overall findings suggested that music therapy leads to significant improvements in patients with autism. However, larger sample sizes and longer durations of study are needed to further validate these results.
